# Introduction to a special issue of *Magnetic Resonance* in honour of Robert Kaptein at the occasion of his 80th birthday

**DOI:** 10.5194/mr-2-465-2021

**Published:** 2021-06-17

**Authors:** Rolf Boelens, Konstantin Ivanov, Jörg Matysik

**Affiliations:** 1 Bijvoet Centre for Biomolecular Research, Utrecht University, 3584 CH Utrecht, the Netherlands; 2 International Tomography Center, Siberian Branch of the Russian Academy of Sciences, Novosibirsk 630090, Russia; 3 Department of Natural Sciences, Novosibirsk State University, Novosibirsk 630090, Russia; 4 Institut für Analytische Chemie, Universität Leipzig, Linnéstraße 3, 04189 Leipzig, Germany

## Abstract

This publication, in honour of Robert Kaptein's 80th birthday, contains contributions from colleagues, many of whom have worked with him, and others who admire his work and have been stimulated by his research. The contributions show current research in biomolecular NMR, spin hyperpolarisation and spin chemistry, including CIDNP (chemically induced dynamic nuclear polarisation), topics to which he has contributed enormously. His proposal of the radical pair mechanism was the birth of the field of spin chemistry, and the laser CIDNP NMR experiment on a protein was a major breakthrough in hyperpolarisation research. He set milestones for biomolecular NMR by developing computational methods for protein structure determination, including restrained molecular dynamics and 3D NMR methodology. With a lac repressor headpiece, he determined one of the first protein structures determined by NMR. His studies of the lac repressor provided the first examples of detailed studies of protein nucleic acid complexes by NMR. This deepened our understanding of protein DNA recognition and led to a molecular model for protein sliding along the DNA. Furthermore, he played a leading role in establishing the cluster of NMR large-scale facilities in Europe. This editorial gives an introduction to the publication and is followed by a biography describing his contributions to magnetic resonance.

## 1 Introduction

The special issue is dedicated to the 80th birthday, on 5 April 2021, of our esteemed colleague, Robert Kaptein, famous for his contributions to spin hyperpolarisation, spin chemistry and biomolecular NMR. In his doctoral research, Kaptein, when trying to explain the perplexing CIDNP (chemically induced dynamic nuclear polarisation) effect, proposed the radical pair mechanism and formulated the famous Kaptein rules, which describe the sign of the CIDNP signals. This development formed the basis for CIDNP as an efficient hyperpolarisation method, providing unique information about elusive short-lived radicals and radical pairs and leading to significant insight in the mechanisms of radical reactions. These results have become one of the cornerstones of an entire new field of science called spin chemistry. Later, Kaptein developed laser photo CIDNP as a selective and sensitive surface probe for studies of proteins and protein interactions in a solution. His interest in spin hyperpolarisation has not been limited to CIDNP; recently, he has also made valuable contributions in understanding the spin dynamics underlying para-hydrogen-induced polarisation (PHIP) and signal amplification by reversible exchange (SABRE)-derived polarisation.

**Figure 1:**
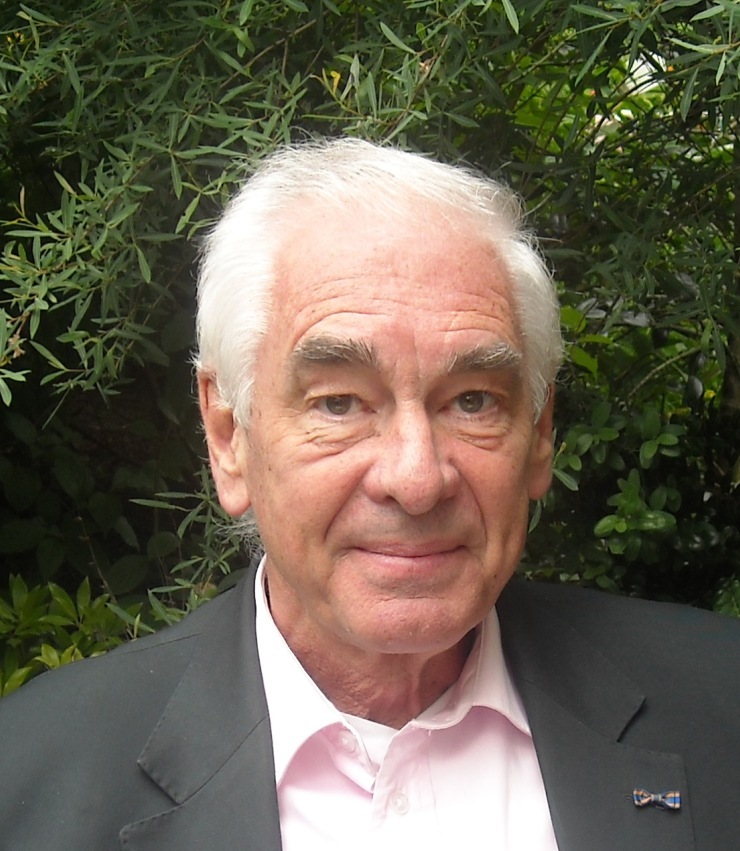
Robert Kaptein today.

Robert Kaptein has also made prominent contributions to the computational and experimental methodology of biomolecular NMR and to the structure and dynamics of gene regulatory proteins and protein DNA complexes. His laboratory developed, among others, non-selective homonuclear 3D NMR, restrained molecular dynamics and methods for relaxation matrix calculations and protein structure validation. The structure of the lac headpiece in 1985 was one of the first protein structures solved by NMR. This was followed by studies on the structure and dynamics of other gene regulatory proteins and protein DNA complexes, such as the glucocorticoid receptor, the Arc repressor and the POU domain of the transcription factor Oct-1. Central to his research has been the studies on the DNA complexes of the lac repressor that not only deepened our understanding of protein DNA recognition but also gave us a molecular model for protein sliding along the DNA. These studies have been of primary importance in establishing NMR as key method for studies on the structure and dynamics of proteins and protein complexes and an important stimulus in developing high-field NMR instrumentation and establishing national and international research infrastructures.

This special issue contains contributions from colleagues, many of whom have worked with Robert Kaptein over the years. The contributions show current research in biomolecular NMR, spin hyperpolarisation and spin chemistry, including CIDNP, all topics to which he has contributed enormously. This special issue nicely illustrates that these topics are still the focus of intense research now. We start with a biography in which we review the contributions of Robert Kaptein to magnetic resonance.

## 2 Biography of Robert Kaptein

This biography focusses on the scientific contributions to spin hyperpolarisation, spin chemistry and biomolecular NMR by Robert Kaptein in the magnetic resonance community. To write this biography, we made use of several historical overviews (Drenth, 2001; de Galan, 2004; Drenth and Verhoeven, 2004; van der Waals and Hilbers, 2004; Kaptein, 2007).

Kaptein studied chemistry at the University of Leiden and started his doctoral research in 1965 in Leiden with Luitzen J. Oosterhoff. The initial plan was to study stable free radicals with NMR. However, in 1967, Bargon and Fischer in Germany and Ward and Lawler in the USA discovered the phenomenon that, in organic chemical radical reactions, intense NMR emission and absorption signals can occur due to the resulting products (Bargon et al., 1967; Ward and Lawler, 1967). A good theoretical interpretation of this so-called CIDNP (chemically induced dynamic nuclear polarisation) effect had not been achieved yet. Kaptein became very interested and gave his thesis research a new direction, with full support by Oosterhoff. At that time, the effect was thought to be related to a dynamic nuclear polarisation (DNP) or Overhauser effect involving free radicals, but this interpretation was not satisfactory. Robert Kaptein – and independently also Gerhard Closs (Closs and Closs, 1969) – was the first to realise that CIDNP formation occurs upon the recombination of radical pairs and provided a clear explanation of the CIDNP effect in three seminal papers (Kaptein and Oosterhoff, 1969a, b; Kaptein, 1972b). The theoretical analysis and results were part of his doctoral thesis in 1971 in Leiden (Kaptein, 1971b). The effects of the CIDNP net and multiplet emissions and absorptions in radical pair reactions could be fully explained by the described radical pair theory. Moreover, the full theory could also be summarised by the appealing so-called Kaptein's rules (Kaptein, 1971a). Using these practical equations, important conclusions could be drawn on the mechanism of radical reactions. His doctoral thesis is a mature and self-contained work that still can be used to learn about radical pair mechanisms and CIDNP. This work was followed by a series of articles with applications to different radical reactions (Kaptein et al., 1971a, b; Kaptein, 1972a, b; Kaptein and den Hollander, 1972; Kaptein et al., 1972, 1973, 1974, 1975; van Leeuwen et al., 1975; den Hollander and Kaptein, 1976).

After his doctorate, driven by the intention to learn about Fourier transform (FT) NMR (a new, fascinating subject in those days), Kaptein went to Varian in Palo Alto, California, for 1 year and worked with Ray Freeman as a postdoc. His work then was dealing with the first ${}^{13}C$ CIDNP detection by FT NMR and developing methods for quantitative ${}^{13}C$ NMR spectroscopy (Freeman et al., 1972). After returning to the Netherlands in 1972, Kaptein joined the KSLA (Royal Shell Laboratory Amsterdam), keeping ties to his colleagues in Leiden. Subsequently, he went back to an academic position.

In 1975, Robert Kaptein became the scientific supervisor of the Netherlands National NMR facility at the University of Groningen with one of the first 360 MHz NMR instruments. He continued his research in the field of CIDNP, which resulted in developing the time-resolved CIDNP method which can be used for a detailed characterisation of the fast reactions of radicals and radical pairs (Hore et al., 1981; Hore and Kaptein, 1982; Hore et al., 1982). Kaptein also developed the photo CIDNP method in which laser-excited flavine dyes form a radical pair with residues at the surface of biomacromolecules (Kaptein et al., 1978). This leads to intense CIDNP effects of the aromatic NMR signals of tyrosine, tryptophan and histidine at the surface of the proteins when accessible for the dye. However, when these surface residues are involved in other interactions due to ligand binding or oligomerisation, the CIDNP signal enhancements are lost. This method enabled detailed functional NMR studies of proteins in a solution and initiated collaborations between Kaptein, in Groningen, with many biochemical groups in the Netherlands and abroad, as summarised in Kaptein (1982). A large number of studies of proteins and biomolecular complexes, using photo CIDNP, followed that allowed them to probe the role of the aromatic residues in these systems. These studies include the complex of the gene-5 protein with DNA with Cees Hilbers (Garssen et al., 1978); phospholipase A2 with Arend-Jan Slotboom, Gerard de Haas and Maarten Egmond (Jansen et al., 1978, 1979; Egmond et al., 1983); ribonuclease with Jaap Beintema (Lenstra et al., 1979); glyceraldehyde-3-phosphate dehydrogenase with Ruud Scheek and Jan Verhoeven (Scheek et al., 1979); colipase with Patrick Cozzone (Canioni et al., 1980); dihydrofolate reductase with Jim Feeney, Gordon Roberts and Angela Gronenborn (Feeney et al., 1980); streptomyces subtilisin inhibitor with Kazuyuki Akasaka (Akasaka et al., 1981); α -lactalbumin with Larry Berliner (Berliner and Kaptein, 1981); flavodoxin with Franz Müller (Moonen et al., 1982); β -endorphin and oligophenols with Lucia Zetta (Zetta et al., 1982, 1988); the interaction of lac repressor with DNA with Heinz Rüterjans (Buck et al., 1983); γ -crystallin with Tom Schleich (Garner et al., 1984); lysozyme with Christina Redfield and Chris Dobson (Redfield et al., 1985); epidermal growth factor and plasminogen kringle domains with Antonio de Marco, Kevin Mayo and Miguel Llinas (de Marco et al., 1986a, b); sea anemone peptides with Ray Norton (Norton et al., 1986); and carbohydrate binding proteins with Hans-Christian Siebert, Hans Gabius and Hans Vliegenthart (Siebert et al., 1995, 1997). But, not only with proteins, the photo CIDNP effect could also be used to probe the accessibility of the aromatic bases in nucleotides and DNA (Kaptein et al., 1979), research that was more recently followed up by Alexandra Yurkovskaya (Morozova et al., 2007, 2008) and in a collaboration with Robert Kaptein (Morozova et al., 2012, 2013). Of course, as with any good rule, exceptions to the Kaptein's rules were also found (Hore et al., 1983), but it is clear that the CIDNP method and the application of photo CIDNP to proteins and protein complexes has been very successful. It also initiated the biomolecular NMR research by Robert Kaptein.

The collaboration with Heinz Rüterjans on the photo CIDNP studies of the lac headpiece, the DNA binding domain of the lac repressor, in a complex with DNA, initiated the multidimensional and structural NMR studies by Robert Kaptein on the lac headpiece and its protein DNA complexes for which he is well known. The preconditions at that time for this research were very favourable; 2D NMR, as developed in Zurich by Richard Ernst, started to be applied to proteins, the method for obtaining the full assignment of protein NMR resonances by 2D NMR had just been developed by Kurt Wüthrich in Zurich, the synthesis of DNA oligonucleotides had just been developed by Jacques van Boom in Leiden, and the development of molecular dynamics simulation techniques of proteins were in full development in Groningen, with Herman Berendsen and Wilfred van Gunsteren. In a short time, Erik Zuiderweg of the group of Robert Kaptein achieved the full assignment of the NMR signals of the lac headpiece in a collaboration with Kurt Wüthrich (Zuiderweg et al., 1983a, b). This was followed by the determination of the 3D structure of the lac headpiece as an early NMR structure, using restrained molecular dynamics for the first time (Kaptein et al., 1985; Zuiderweg et al., 1985). Next, analogous to the sequential assignment procedure for proteins, a sequential NMR assignment method was developed for assigning the resonances in DNA using 2D NOE (nuclear Overhauser effect) spectra (Scheek et al., 1983, 1984; Boelens et al., 1985). Soon thereafter, a first model of a protein DNA complex by NMR was presented of the lac headpiece in a complex with the left half of the lac operator (Boelens et al., 1987). This model showed that the recognition helix of the lac headpiece would bind in the major groove of DNA, in line with biochemical data, but surprisingly, the orientation of the helix was 180 ${}^{\circ }$ opposite to what had been predicted on the basis of the X-ray structures of the dimeric Cro and lambda repressors (Ohlendorf et al., 1982; Pabo and Lewis, 1982). Essentially, this model was mainly based on two firm intermolecular NOEs, but the fact that this model was correct became evident from subsequent biochemical analysis of lac repressor DNA complexes by Benno Müller-Hill (Lehming et al., 1987, 1988) and was also demonstrated in detailed studies with highly optimised headpiece DNA complexes that showed a large number of intermolecular NOEs (Chuprina et al., 1993) and by subsequent studies of dimers of the lac headpiece bound to full 22 bp lac operators (Lamerichs et al., 1989; Spronk et al., 1996, 1999; Kalodimos et al., 2002). These studies allowed a detailed understanding of the precise recognition of the operator by the lac repressor (20 bp in a $4.6\times 10^{6}$ bp *E. coli* genome) through a multitude of intermolecular interactions. A further highlight was the study of the complex of a lac headpiece dimer with non-specific DNA (Kalodimos et al., 2004a, b). It turned out that the topologies of the specific and non-specific DNA complexes are very similar, with the recognition helices, in both cases, in the major groove of DNA. Crucial differences, however, were that the recognition helix in the non-specific complex was not as deeply positioned in the major groove, many side chains that formed hydrogen bonds in the major groove of the specific complex were redirected to the DNA phosphate backbone, and the hinge helices that stabilised the specific lac operator complexes were absent in the non-specific complex. The non-specific DNA binding also provided a model for 1D sliding of the lac repressor along the DNA, which explains the fast approach of the lac repressor towards the lac operator site (von Hippel and Berg, 1989; Kalodimos et al., 2004b). In fact, this sliding along the DNA phosphate backbone could even be directly studied by NMR (Loth et al., 2013).

In parallel to these biomolecular NMR studies, Kaptein's group also made visible contributions to NMR development. In addition to the photo CIDNP method as discussed above, the group developed, in collaboration with Wilfred van Gunsteren, restrained molecular dynamics for structure determination of biomolecules (van Gunsteren et al., 1984), methodology that can still be found in programmes such as Xplor and CNS (Brünger, 1992; Brünger et al., 1998). An iterative relaxation matrix approach (IRMA) using restrained molecular dynamics (MDs) was developed that allowed one to obtain precise distances from the 2D NOE spectra of biomolecules (Boelens et al., 1988, 1989a) for structure calculations, including DNA oligomers (Koning et al., 1991). Later, when combined with ensemble averaging, this approach allowed protein structure determination with accurate side chain geometries (Bonvin et al., 1994). Kaptein also made important contributions to the validation of structures determined by NMR. In collaboration with Kaptein, the group of Janet Thornton (UK) developed the widely used programme PROCHECK-NMR that enabled one to precisely analyse the violations of NMR restraints used in the structure calculations (Laskowski et al., 1996). A highly visible structural analysis by Kaptein's group was also the recalculation of more than 500 NMR structures in the Protein Data Bank (PDB), using restraints deposited in the Biological Magnetic Resonance Data Bank (BMRB), a study that was a cornerstone for NMR structure validation (Nederveen et al., 2005). For many years, Robert Kaptein chaired the NMR task force that advised the worldwide PDB and made the recommendations for nomenclature, representation of NMR structures and deposition of experimental NMR data in the databases for NMR of RCSB (PDB in the USA), European Bioinformatics Institute (EBI) and BMRB (Markley et al., 1998), which further indicates the crucial role that Kaptein has played in this field. Other methodological developments by Kaptein's group were contributions to the development of 3D NMR. In a first paper of his group, 3D J-resolved NMR was demonstrated (Vuister and Boelens, 1987), which led to the development of 3D COSY-COSY and COSY-NOESY by Christian Griesinger and Richard Ernst (Griesinger et al., 1987) and then to the first non-selective 3D NOESY-TOCSY and 3D NOESY-NOESY experiments in the group of Kaptein (Vuister et al., 1988; Boelens et al., 1989b). This paved the way for the highly successful heteronuclear 3D NMR developed later on in the USA (Marion et al., 1989; Zuiderweg and Fesik, 1989).

The visible research on the lac repressor research triggered a series of collaborative NMR studies of other DNA binding proteins with models for DNA binding. Examples are the Arc and Mnt repressors, which use a β sheet for DNA recognition (Breg et al., 1990; Burgering et al., 1994), a collaboration with Robert Sauer of MIT in Boston; the DNA binding domain of the glucocorticoid hormone receptor with Keith Yamamoto (San Francisco) and Jan-Ake Gustaffson (Stockholm; Härd et al., 1990); the DNA binding domain of the retinoic acid hormone receptor with Paul van der Saag (Knegtel et al., 1993); the POU domain of the Oct-1 transcription factor with Peter van der Vliet in Utrecht (Dekker et al., 1993); the bacterial HU protein with Keith Wilson and Costas Vorgias of the European Molecular Biology Laboratory (EMBL; Vis et al., 1995); a model for the binding of ribosomal initiation factor IF1 to the 30S ribosomal subunit with Claudio Gualerzi in Camerino, Italy (Sette et al., 1997); the human DNA repair complex ERCC1/XPF with Jan Hoeijmakers in Rotterdam (Tripsianes et al., 2005); and the N- and C-terminal domains of HIV integrase with Ronald Plasterk of the Netherlands Cancer Institute in Amsterdam (Eijkelenboom et al., 1995, 2000). Other structures determined were that of the complex of phospholipase A2 on a micellar surface and an inhibitor, with Bert Verweij in Utrecht (van den Berg et al., 1995a, b), and that of the complex of the lantibiotic nisin with lipid II, with Ben de Kruijff in Utrecht (Hsu et al., 2004).

The NMR studies of the photoactive yellow protein (PYP) and the blue light using FAD (BLUF) domain of AppA, a collaboration with the group of Klaas Hellingwerf in Amsterdam, combined the extensive expertise in Kaptein's group of laser light excitation in NMR with studies on protein structure and dynamics. After identifying the chromophore of PYP (Hoff et al., 1994), the group studied the photocycle intermediates of PYP. It could be shown that the light-excited state of PYP is highly dynamic and partially unfolds (Rubinstenn et al., 1998), a result that was in marked contrast to that obtained by X-ray crystallography. Whereas the structure of the ground state of wild-type PYP could be fully characterised by NMR (Düx et al., 1998; Craven et al., 2000), detailed analysis for the light-excited state turned out to be difficult since it was rapidly converted back to its ground state (Rubinstenn et al., 1998). Shortly after, however, it was found that the light-excited state of Δ 25 PYP missing 25 N-terminal residues was much longer lived (van der Horst et al., 2001). This enabled a full analysis of the structure, folding and dynamics of Δ 25 PYP in the ground and light-excited state (Bernard et al., 2005). It was found that, after light excitation of Δ 25 PYP, a large N-terminal part of the protein largely unfolds, which settled NMR as a powerful tool for studying the photocycle intermediates of these photoreceptors.

After his official retirement in 2006, Robert Kaptein remained active in the field of bio NMR and spin hyperpolarisation. An important part of these activities was his Russian adventure. In 2011, Robert Kaptein was appointed as a leading scientist in the so-called Megagrant programme of the Russian Ministry of Science and Education, aimed at attracting leading researchers to Russia to initiate and lead cutting-edge research activities. The application had been prepared together with a team from Novosibirsk, led by Renad Sagdeev. The key participants from the Russian side, representing the Novosibirsk State University and International Tomography Center, were Alexandra Yurkovskaya, Nikita Lukzen, Igor Koptyug, Yuri Tsentalovich and Konstantin Ivanov. As usual, Kaptein's efforts have helped to improve research infrastructure (two modern NMR spectrometers could be purchased through the Megagrant). Thereafter, 2 years of research on spin hyperpolarisation and NMR followed, with frequent and extended stays of Kaptein in Novosibirsk (2012–2013). This highly stimulated progress in understanding the peculiarities of polarisation formation and polarisation transfer in experiments with parahydrogen. Notably, ideas of using level anti-crossings to describe polarisation transfer were developed during that period (Ivanov et al., 2013; Pravdivtsev et al., 2013a). These ideas were particularly rewarding in the case of SABRE (signal amplification by reversible exchange), which was, in those days, a new method for exploiting parahydrogen for NMR signal enhancement (Pravdivtsev et al., 2015). Robert Kaptein stimulated interest for this method in the Novosibirsk team, which later resulted in describing the magnetic field dependence of polarisation and also – as in the case of CIDNP – in formulating polarisation sign rules (Pravdivtsev et al., 2013b). The description of the role of level anti-crossings can be found in a review paper (Ivanov et al., 2014).

For his excellent research, Robert Kaptein was awarded the Gold Medal of the Royal Dutch Chemical Society and the Jean-Servais Stas Medal of the Société Chimique de Belgique in 1971 and the Holleman award of the Royal Netherlands Academy of Arts and Sciences in 1985. He was elected a member of the Royal Netherlands Academy of Arts and Sciences in 1989, a member of EMBO in 1991 and, in 1997, an honorary member of the National Magnetic Resonance Society of India. In 1980, he was appointed professor in Chemistry at the University of Groningen and in 1987 at the University of Utrecht. He has been research director of the Bijvoet Center for Biomolecular Research from 2000 to 2006, Secretary General of the Royal Dutch Academy of Sciences from 2002 to 2008, a member of various Scientific advisory boards such as EMBL, EBI, and ENS in Paris. He was a member and is a fellow of the International Society of Magnetic Resonance (ISMAR) council, chaired the International Council on Magnetic Resonance in Biological Systems (ICMRBS) and was a member of the advisory board of the PDB.

As son of a teacher, Robert Kaptein had teaching in his genes. In addition to being an outstanding researcher, he was an excellent teacher. Still in Groningen, Kaptein was elected by students as one of their best teachers. These teaching skills came also into practice in teaching NMR to students at Utrecht. Together with Cees Hilbers in Nijmegen, Kaptein initiated advanced courses in biomolecular NMR, which trained many students and postdocs at that time in the Netherlands and which highly contributed to the development of NMR in the Netherlands. In total, Robert Kaptein trained 43 doctoral students, some already in Groningen but most of them in the NMR laboratory in Utrecht, and also hosted many postdocs and guest researchers. Many of them became professors thereafter. Together with Christian Griesinger and Hartmut Oschkinat, he initiated, in 1995, the EMBO course of multidimensional NMR in structural biology that was held for many years in Il Ciocco in Italy and trained many young scientists in NMR, who are currently active researchers in Europe and abroad. Robert Kaptein is an active member of the European NMR family. Next to being an outstanding scientist, he was an excellent organiser. Not only has he established a world-class biomolecular NMR lab in Utrecht but for several years he has been the director of the Bijvoet Center for Biomolecular Research and Secretary General of the Royal Netherlands Academy of Arts and Sciences (KNAW). From its start in 1991, Kaptein has been and still is associate editor of the *Journal of Biomolecular NMR*. He has also been editor or associate editor of other journals, including the *Journal of Magnetic Resonance* and *Magnetic Resonance, Biopolymers, Structure* and also the editor of series such as *Focus on Structural Biology* and *Comprehensive Biomolecular Nuclear Magnetic Resonance*.

An important, often underrated, aspect of NMR is organising and maintaining its infrastructure. Of course, many elements come into play, namely the skills and success of the researcher and willingness of the environment to breed, embed and support such infrastructure, and all at the same time. What turned out not to be possible in Groningen became a big success in Utrecht. When Kaptein was attracted to Utrecht in 1987, a 500 MHz NMR was funded by the university, the 360 MHz could be moved from Groningen, and a focussed research group could be built up. A few years later, the group could install a state-of-the-art 600 MHz NMR. As one of the first, the group could install a 750 MHz NMR in 1994, and Utrecht became a national and European high-field NMR facility and expanded with an additional 500 and 600 MHz instrument. In 2001, a state-of-the-art 900 MHz NMR could be acquired, which was installed in a new building in 2003, the Nicolaas Bloembergen building, which houses all instruments of the Utrecht NMR group and which will also be the base for a 1.2 GHz NMR, planned to be installed in Utrecht in 2021. Together with Ivano Bertini (later Claudio Luchinat and Lucia Banci) in Florence, Italy, and Heinz Rüterjans (and later Harald Schwalbe) in Frankfurt, Germany, these three NMR facilities have, since 1994, formed the core of a cluster of European NMR facilities open to researchers in Europe and worldwide. Robert Kaptein has played a pivotal role in initiating, organising and maintaining these infrastructures.

## References

[bib1.bib1] Akasaka K, Fujii S, Kaptein R (1981). Exposure of Aromatic Residues of Streptomyces Subtilisin Inhibitor. A Photo-CIDNP study. J Biochem-Tokyo.

[bib1.bib2] Bargon J, Fischer H, Johnson U (1967). Kernresonanz-Emissionslinien während rascher Radikalreaktionen. 1. Aufnahmeverfahren und Beispiele. Z Naturforsch.

[bib1.bib3] Berliner LJ, Kaptein R (1981). NMR Characterization of Aromatic Residues of 
α
-Lactalbumins. Laser Photo Chemically Induced Dynamic Nuclear Polarization Nuclear Magnetic Resonance Studies of Surface Exposure. Biochemistry.

[bib1.bib4] Bernard C, Houben K, Derix NM, Marks D, van der Horst MA, Hellingwerf KJ, Boelens R, Kaptein R, van Nuland NAJ (2005). The solution structure of a transient photoreceptor intermediate: 
Δ25
 photoactive yellow protein. Structure.

[bib1.bib5] Boelens R, Scheek RM, Dijkstra K, Kaptein R (1985). Sequential Assignment of Imino- and Amino-Proton Resonances in 
${}^{1}H$
 NMR Spectra of Oligonucleotides by Two-Dimensional NMR Spectroscopy. Application to a *lac* Operator Fragment. J Magn Reson.

[bib1.bib6] Boelens R, Scheek RM, van Boom JH, Kaptein R (1987). Complex of *lac* repressor Headpiece with a 14 base-pair *lac* operator fragment studied by two-dimensional nuclear magnetic resonance. J Mol Biol.

[bib1.bib7] Boelens R, Koning TMG, Kaptein R (1988). Determination of biomolecular structures from proton-proton NOE's using a relaxation matrix approach. J Mol Struct.

[bib1.bib8] Boelens R, Koning TMG, van der Marel GA, van Boom JH, Kaptein R (1989). Iterative procedure for structure determination from proton-proton NOE's using a full relaxation matrix approach. Application to a DNA octamer. J Magn Reson.

[bib1.bib9] Boelens R, Vuister GW, Koning TMG, Kaptein R (1989). Observation of spin-diffusion in biomolecules by three-dimensional NOE-NOE spectroscopy. J Am Chem Soc.

[bib1.bib10] Bonvin AMJJ, Vis H, Breg JN, Burgering MJM, Boelens R, Kaptein R (1994). NMR solution structure of the Arc repressor using relaxation matrix calculations. J Mol Biol.

[bib1.bib11] Breg JN, van Opheusden JHJ, Burgering MJM, Boelens R, Kaptein R (1990). The structure of Arc repressor in solution. A family of 
β
-sheet DNA-binding proteins. Nature.

[bib1.bib12] Brünger AT (1992). X-PLOR, Version 3.1, A System for X-ray Crystallography and NMR.

[bib1.bib13] Brünger AT, Adams PD, Clore GM, DeLano WL, Gros P, Grosse-Kunstleve RW, Jiang JS, Kuszewski J, Nilges M, Pannu NS, Read RJ, Rice LM, Simonson T, Warren GL (1998). Crystallography & NMR system: A new software suite for macromolecular structure determination. Acta Crystallogr D.

[bib1.bib14] Buck F, Hahn KD, Zemann W, Rüterjans H, Sadler JR, Beyreuther K, Kaptein R, Scheek R, Hull WE (1983). NMR Study of the Interaction between the *lac* Repressor and the *lac* Operator. Eur J Biochem.

[bib1.bib15] Burgering MJM, Boelens R, Gilbert DE, Breg JN, Knight KL, Sauer RT, Kaptein R (1994). Solution Structure of Dimeric Mnt Repressor (1-76). Biochemistry.

[bib1.bib16] Canioni P, Cozzone PJ, Kaptein R (1980). 360 MHz Laser Photo-CIDNP of Porcine Pancreatic Colipase A. A Study of the Aromatic Surface Residues. FEBS Lett.

[bib1.bib17] Chuprina VP, Rullmann JAC, Lamerichs RMJN, van Boom JH, Boelens R, Kaptein R (1993). Structure of the Complex of *lac* Repressor Headpiece and an 11 Base Pair Half-Operator determined by NMR Spectroscopy and Restrained Molecular Dynamics. J Mol Biol.

[bib1.bib18] Closs GL, Closs LE (1969). Induced dynamic nuclear spin polarization in
reactions of photochemically and thermally generated triplet
diphenylmethylene. J Am Chem Soc.

[bib1.bib19] Craven CJ, Derix NM, Hendriks J, Boelens R, Hellingwerf KJ, Kaptein R (2000). Probing the nature of the blue-shifted intermediate of photoactive yellow protein in solation by NMR: Hydrogen-deuterium exchange data and pH studies. Biochemistry.

[bib1.bib20] de Galan L (2004). De geschiedenis van de scheikunde in Nederland 3. De ontwikkeling van de chemie van 1945 tot het begin van de jaren tachtig.

[bib1.bib21] Dekker N, Cox M, Boelens R, Verrijzer CP, van der Vliet PC, Kaptein R (1993). Solution structure of the POU-specific DNA binding domain of Oct-1. Nature.

[bib1.bib22] de Marco A, Mayo KH, Bartolotti F, Scalia S, Menegatti E, Kaptein R (1986). Proton NMR and photo-CIDNP studies of peptide fragments obtained by controlled proteolysis of mouse epidermal growth factor. J Biol Chem.

[bib1.bib23] de Marco A, Zetta L, Petros AM, Llinas M, Boelens R, Kaptein R (1986). Kringle 4 from human plasminogen: A proton Magnetic Resonance study via two-dimensional photochemically induced dynamic nuclear polarization spectroscopy. Biochemistry.

[bib1.bib24] den Hollander JA, Kaptein R (1976). Radical Pair Substitution in CIDNP. Spin-Uncorrelated Geminate Radical Pairs. Chem Phys Lett.

[bib1.bib25] Drenth W (2001). 120 jaar fysisch-organische chemie in Nederland 1874–1994.

[bib1.bib26] Drenth W, Verhoeven JW (2004). De geschiedenis van de scheikunde in Nederland 3. De ontwikkeling van de chemie van 1945 tot het begin van de jaren tachtig.

[bib1.bib27] Düx P, Rubinstenn G, Vuister GW, Boelens R, Mulder FAA, Hård K, Hoff WD, Kroon AR, Crielaard W, Hellingwerf KJ, Kaptein R (1998). Solution structure and backbone dynamics of the photoactive yellow protein. Biochemistry.

[bib1.bib28] Egmond MR, Hore PJ, Kaptein R (1983). Photo-CIDNP 
${}^{1}H$
-NMR Studies of Bovine Pancreactic Phospholipase A
${}_{2}$
 and its Zymogen. Biochim Biophys Acta.

[bib1.bib29] Eijkelenboom APAM, Lutzke RAP, Boelens R, Plasterk RHA, Kaptein R, Hård K (1995). The DNA-Binding domain of HIV-1 Iintegrase has an SH3-like fold. Nat Struct Biol.

[bib1.bib30] Eijkelenboom APAM, van den Ent FMI, Wechselberger R, Plasterk RHA, Kaptein R, Boelens R (2000). Refined solution structure of the dimeric N-terminal HHCC domain of HIV-2 integrase. J Biomol NMR.

[bib1.bib31] Feeney J, Roberts GCK, Kaptein R, Birdsall B, Gronenborn A, Burgen ASV (1980). Photo-CIDNP Studies of the Influence of Ligand Binding on the Surface Accessibility of Aromatic Residues in Dihydrofolate Reductase. Biochemistry.

[bib1.bib32] Freeman R, Hill HDW, Kaptein R (1972). Proton-Decoupled NMR Spectra of Carbon-13 With the Nuclear Overhauser Effect Suppressed. J Magn Reson.

[bib1.bib33] Garner WH, Spector A, Schleich T, Kaptein R (1984). Determination of the solvent accessibility of specific aromatic residues in gamma-crystallin by Photo-CIDNP NMR measurements. Curr Eye Res.

[bib1.bib34] Garssen GJ, Kaptein R, Schoenmakers JGG, Hilbers CW (1978). A photo-CIDNP study of the interaction of oligonucleotides with gene-5 protein of bacteriophage M13. P Natl Acad Sci USA.

[bib1.bib35] Griesinger C, Sørensen OW, Ernst RR (1987). A practical approach to three-dimensional NMR spectroscopy. J Magn Reson.

[bib1.bib36] Härd T, Kellenbach E, Boelens R, Maler BA, Dahlman K, Freedman LP, Carlstedt-Duke J, Yamamoto KR, Gustafsson JA, Kaptein R (1990). Solution Structure of the Glucocorticoid Receptor DNA-Binding Domain. Science.

[bib1.bib37] Hoff WD, Düx P, Hård K, de Vreese B, Nugteren-Roodzant IM, Crielaard W, Boelens R, Kaptein R, van Beeumen J, Hellingwerf KJ (1994). Thiol ester-linked p-coumaric acid as a new photoactive cofactor in a rhodopsin-like protein. Biochemistry.

[bib1.bib38] Hore PJ, Kaptein R (1982). Photo-CIDNP of Biological Molecules using Continuous Wave and Time-Resolved Methods. ACS Symp Ser.

[bib1.bib39] Hore PJ, Zuiderweg ERP, Kaptein R, Dijkstra K (1981). Flash Photolysis NMR. CIDNP Time Dependence in Cyclic Photochemical Reactions. Chem Phys Lett.

[bib1.bib40] Hore PJ, Volbeda A, Dijkstra K, Kaptein R (1982). Photo-Reduction of Flavin by NADH – a flash-photolysis photo-CIDNP study. J Am Chem Soc.

[bib1.bib41] Hore PJ, Stob S, Kemmink J, Kaptein R (1983). An Exception to the CIDNP Sign Rules. Chem Phys Lett.

[bib1.bib42] Hsu STD, Breukink E, Tischenko E, Lutters MAG, de Kruijff B, Kaptein R, Bonvin AMJJ, van Nuland NAJ (2004). The nisin-lipid II complex reveals a pyrophosphate cage that provides a blueprint for novel antibiotics. Nat Struct Mol Biol.

[bib1.bib43] Ivanov KL, Stass DV, Kalneus EV, Kaptein R, Lukzen NN (2013). Theoretical Treatment of Degenerate Electron Exchange and Dimerization in Spin Dynamics of Radical Ion Pairs as Observed by Magnetic Field Effects. Appl Magn Reson.

[bib1.bib44] Ivanov KL, Pravdivtsev AN, Yurkovskaya AV, Vieth HM, Kaptein R (2014). The role of level anti-crossings in nuclear spin hyperpolarization. Prog Nucl Mag Res Sp.

[bib1.bib45] Jansen EHJM, Meyer H, de Haas GH, Kaptein R (1978). A Photo-CIDNP Study of Pancreatic Phospholipase A
${}_{2}$
. NMR Assignments of some Aromatic Residues. J Biol Chem.

[bib1.bib46] Jansen EHJM, van Scharrenburg GJM, Slotboom AJ, de Haas GH, Kaptein R (1979). A 360 MHz Photo-CIDNP Study of Bovine Pancreatic Phospholipase A
${}_{2}$
. Observation of a pH Dependent Conformational Change. J Am Chem Soc.

[bib1.bib47] Kalodimos CG, Bonvin AMJJ, Salinas RK, Wechselberger R, Boelens R, Kaptein R (2002). Plasticity in protein-DNA recognition: *lac* repressor interacts with its natural operator *O1* through alternative conformations of its DNA-binding domain. EMBO J.

[bib1.bib48] Kalodimos CG, Biris N, Bonvin AMJJ, Levandoski MM, Guennuegues M, Boelens R, Kaptein R (2004). Structure and flexibility adaptation in nonspecific and specific protein-DNA complexes. Science.

[bib1.bib49] Kalodimos CG, Boelens R, Kaptein R (2004). Toward an integrated model of protein-DNA recognition as inferred from NMR studies on the Lac repressor system. Chem Rev.

[bib1.bib50] Kaptein R (1971). Simple Rules for Chemically Induced Dynamic Nuclear Polarization. J Chem Soc Chem Comm.

[bib1.bib51] Kaptein R (1971). Chemically Induced Dynamic Nuclear Polarization [PhD thesis].

[bib1.bib52] Kaptein R (1972). Chemically Induced Dynamic Nuclear Polarization. IX. Reactions competitive with germinate recombination of radical pairs. J Am Chem Soc.

[bib1.bib53] Kaptein R (1972). Chemically Induced Dynamic Nuclear Polarization. VIII. Spin Dynamics and Diffusion of Radical Pairs. J Am Chem Soc.

[bib1.bib54] Kaptein R, Berliner LJ, Rueben J (1982). Photo-CIDNP Studies of Proteins. Biological Magnetic Resonance, vol 4.

[bib1.bib55] Kaptein R, Harris RK, Wasylishen R (2007). Encyclopedia of Magnetic Resonance.

[bib1.bib56] Kaptein R, den Hollander JA (1972). Chemically Induced Dynamic Nuclear Polarization. X. On the Magnetic Field Dependence. J Am Chem Soc.

[bib1.bib57] Kaptein R, Oosterhoff LJ (1969). Chemically induced dynamic nuclear polarization. III. (anomalous multiplets of radical coupling and disproportionation products). Chem Phys Lett.

[bib1.bib58] Kaptein R, Oosterhoff LJ (1969). Chemically induced dynamic nuclear polarization. II. (Relation with anomalous ESR spectra). Chem Phys Lett.

[bib1.bib59] Kaptein R, Fráter-Schröder M, Oosterhoff LJ (1971). Chemically Induced Dynamic Nuclear Polarization. V. NMR Enhancements in Biradical Products. Chem Phys Lett.

[bib1.bib60] Kaptein R, Verheus FW, Oosterhoff LJ (1971). Chemically Induced Dynamic Nuclear Polarization. VI. Sign Reversal of the Polarization in the Reaction of Isobutyryl Peroxide with Bromotrichloromethane. J Chem Soc Chem Comm.

[bib1.bib61] Kaptein R, Brokken-Zijp J, de Kanter FJJ (1972). Chemically Induced Dynamic Nuclear Polarization. XI. Thermal Decomposition of Acetyl Peroxide. J Am Chem Soc.

[bib1.bib62] Kaptein R, Freeman R, Hill HDW, Bargon J (1973). Carbon-13 CIDNP in the Reversible Addition of Pentafluorobenzoyloxy Radicals to Chlorobenzene. J Chem Soc Chem Comm.

[bib1.bib63] Kaptein R, Freeman R, Hill HDW (1974). Carbon-13 CIDNP from Biradicals in the Photolysis of Cyclic Ketones. Chem Phys Lett.

[bib1.bib64] Kaptein R, van Leeuwen PWNM, Huis R (1975). CIDNP Study of Homolytic Substitution (
$S_{H}2$
) Reactions at Metal Centres. J Chem Soc Chem Comm.

[bib1.bib65] Kaptein R, Dijkstra K, Nicolay K (1978). Laser Photo-CIDNP as a Surface Probe for Proteins in Solution. Nature.

[bib1.bib66] Kaptein R, Nicolay K, Dijkstra K (1979). Photo-CIDNP in Nucleic Acid Bases and Nucleotides. J Chem Soc Chem Comm.

[bib1.bib67] Kaptein R, Zuiderweg ERP, Scheek RM, Boelens R, van Gunsteren WF (1985). A Protein Structure from Nuclear Magnetic Resonance Data: *lac* Repressor Headpiece. J Mol Biol.

[bib1.bib68] Knegtel RMA, Katahira M, Schilthuis JG, Bonvin AMJJ, Boelens R, Eib D, van der Saag PT, Kaptein R (1993). The solution structure of the human retinoic acid receptor-beta DNA-binding domain. J Biomol NMR.

[bib1.bib69] Koning TMG, Boelens R, van der Marel GA, van Boom JH, Kaptein R (1991). Structure determination of a DNA octamer in solution by NMR spectroscopy. The effect of fast local motions. Biochemistry.

[bib1.bib70] Lamerichs RMJN, Boelens R, van der Marel GA, van Boom JH, Kaptein R, Buck F, Fera B, Rüterjans H (1989). Proton NMR study of a complex between the lac repressor headpiece and a 22 base pair symmetric lac operator. Biochemistry.

[bib1.bib71] Laskowski RA, Rullmann JAC, MacArthur MW, Kaptein R, Thornton JM (1996). AQUA and PROCHECK-NMR: Programs for checking the quality of protein structures solved by NMR. J Biomol NMR.

[bib1.bib72] Lehming N, Sartorius J, Niemöller M, Genenger G, von Wilcken-Bergmann B, Müller-Hill B (1987). The interaction of the recognition helix of lac repressor with lac operator. EMBO J.

[bib1.bib73] Lehming N, Sartorius J, Oehler S, von Wilcken-Bergmann B, Müller-Hill B (1988). Recognition helices of lac and lambda repressor are oriented in opposite directions and recognize similar DNA sequences. P Natl Acad Sci USA.

[bib1.bib74] Lenstra JA, Bolscher BGJM, Stob S, Beintema JJ, Kaptein R (1979). The Aromatic Residues of Bovine Ribonuclease Studied by 
${}^{1}H$
 Nuclear Magnetic Resonance. Eur J Biochem.

[bib1.bib75] Loth K, Gnida M, Romanuka J, Kaptein R, Boelens R (2013). Sliding and target location of DNA-binding proteins:an NMR view of the lac repressor system. J Biomol NMR.

[bib1.bib76] Marion D, Driscoll PC, Kay LE, Wingfield PT, Bax A, Gronenborn AM, Clore GM (1989). Overcoming the overlap problem in the assignment of proton NMR spectra of larger proteins by use of three-dimensional heteronuclear proton-nitrogen-15 Hartmann-Hahn-multiple quantum coherence and nuclear Overhauser-multiple quantum coherence spectroscopy: application to interleukin 1.beta. Biochemistry.

[bib1.bib77] Markley JL, Bax A, Arata Y, Hilbers CW, Kaptein R, Sykes BD, Wright PE, Wüthrich K (1998). Recommendations for the presentation of NMR structures of proteins and nucleic acids – (IUPAC Recommendations 1998). Pure Appl Chem.

[bib1.bib78] Moonen CTW, Hore PJ, Müller F, Kaptein R, Mayhew SG (1982). A Photo-CIDNP Study of the active sites of Megashaera elsdenii and Clostridium MP flavodoxins. FEBS Lett.

[bib1.bib79] Morozova OB, Kiryutin AS, Sagdeev RZ, Yurkovskaya AV (2007). Electron transfer between guanosine radical and amino acids in aqueous solution. 1. Reduction of guanosine radical by tyrosine. J Phys Chem B.

[bib1.bib80] Morozova OB, Kiryutin AS, Yurkovskaya AV (2008). Electron transfer between guanosine radicals and amino acids in aqueous solution. II. Reduction of guanosine radicals by tryptophan. J Phys Chem B.

[bib1.bib81] Morozova OB, Kaptein R, Yurkovskaya AV (2012). Reduction of guanosyl radical by cysteine and cysteine-glycine studied by time-resolved CIDNP. J Phys Chem B.

[bib1.bib82] Morozova OB, Kaptein R, Sagdeev RZ, Yurkovskaya AV (2013). Reduction of Guanosyl Radicals in Reactions with Proteins Studied by TR-CIDNP. Appl Magn Reson.

[bib1.bib83] Nederveen AJ, Doreleijers JF, Vranken W, Miller Z, Spronk CAEM, Nabuurs SB, Güntert P, Livny M, Markley JL, Nilges M, Ulrich EL, Kaptein R, Bonvin AMJJ (2005). RECOORD: A recalculated coordinate database of 500+ proteins from the PDB using restraints from the BioMagResBank. Proteins.

[bib1.bib84] Norton RS, Beress L, Stob S, Boelens R, Kaptein R (1986). Photochemically induced dynamic nuclear polarisation NMR study of the aromatic residues of sea-anemone polypeptide cardiac stimulants. Eur J Biochem.

[bib1.bib85] Ohlendorf DH, Anderson WF, Fisher RG, Takeda Y, Matthews BW (1982). The molecular basis of DNA-protein recognition inferred from the structure of cro repressor. Nature.

[bib1.bib86] Pabo CO, Lewis M (1982). The operator-binding domain of lambda repressor: structure and DNA recognition. Nature.

[bib1.bib87] Pravdivtsev AN, Ivanov KL, Kaptein R, Yurkovskaya AV (2013). Theoretical Study of Dipolar Relaxation of Coupled Nuclear Spins at Variable Magnetic Field. Appl Magn Reson.

[bib1.bib88] Pravdivtsev AN, Yurkovskaya AV, Kaptein R, Miesel K, Vieth HM, Ivanov KL (2013). Exploiting level anti-crossings for efficient and selective transfer of hyperpolarization in coupled nuclear spin systems. Phys Chem Chem Phys.

[bib1.bib89] Pravdivtsev AN, Ivanov KL, Yurkovskaya AV, Petrov PA, Limbach HH, Kaptein R, Vieth HM (2015). Spin polarization transfer mechanisms of SABRE: A magnetic field dependent study. J Magn Reson.

[bib1.bib90] Redfield C, Dobson CM, Scheek RM, Stob S, Kaptein R (1985). Surface accessibility of aromatic residues in human lysozyme using photochemically induced dynamic nuclear polarization NMR spectroscopy. FEBS Lett.

[bib1.bib91] Rubinstenn G, Vuister GW, Mulder FAA, Dux PE, Boelens R, Hellingwerf KJ, Kaptein R (1998). Structural and dynamic changes of photoactive yellow protein during its photocycle in solution. Nat Struct Biol.

[bib1.bib92] Scheek RM, Kaptein R, Verhoeven JW (1979). Resolution of specific histidine resonances in the 360 MHz 
${}^{1}H$
 NMR spectrum of glyceraldehyde-3-phosphate dehydrogenase, a 145 000 molecular weight protein, by photo-CIDNP. FEBS Lett.

[bib1.bib93] Scheek RM, Russo N, Boelens R, Kaptein R (1983). Sequential resonance assignments in DNA proton NMR spectra by two-dimensional NOE spectroscopy. J Am Chem Soc.

[bib1.bib94] Scheek RM, Boelens R, Russo N, van Boom JH, Kaptein R (1984). Sequential resonance assignments in proton NMR spectra of oligonucleotides by two-dimensional NMR spectroscopy. Biochemistry.

[bib1.bib95] Sette M, van Tilborg PJA, Spurio R, Kaptein R, Paci M, Gualerzi CO, Boelens R (1997). The structure of the translational initiation factor IF1 from *E. coli* contains an oligomer-binding motif. EMBO J.

[bib1.bib96] Siebert HC, Andre S, Reuter G, Gabius HJ, Kaptein R, Vliegenthart JFG (1995). Effect of enzymatic desialylation of human serum amyloid P component on surface exposure of laser photo CIDNP (chemically induced dynamic nuclear polarization) – reactive histidine, tryptophan and tyrosine residues. FEBS Lett.

[bib1.bib97] Siebert HC, Kaptein R, Beintema JJ, Soedjanaatmadja UM, Wright CS, Rice A, Kleineidam RG, Kruse S, Schauer R, Pouwels PJW, Kamerling JP, Gabius HJ, Vliegenthart JFG (1997). Carbohydrate-protein interaction studies by laser photo CIDNP NMR methods. Glycoconjugate J.

[bib1.bib98] Spronk CAEM, Slijper M, van Boom JH, Kaptein R, Boelens R (1996). Formation of the hinge helix in the lac repressor is induced upon binding to the lac operator. Nat Struct Biol.

[bib1.bib99] Spronk CAEM, Folkers GE, Noordman AMGW, Wechselberger R, van den Brink N, Boelens R, Kaptein R (1999). Hinge-helix formation and DNA bending in various *lac* repressor-operator complexes. The EMBO J.

[bib1.bib100] Tripsianes K, Folkers G, AB E, Das D, Odijk H, Jaspers NGJ, Hoeijmakers JHJ, Kaptein R, Boelens R (2005). The structure of the human ERCC1/XPF interaction domains reveals a complementary role for the two proteins in nucleotide excision repair. Structure.

[bib1.bib101] van den Berg B, Tessari M, Boelens R, Dijkman R, de Haas GH, Kaptein R, Verheij HM (1995). NMR structures of phospholipase A
${}_{2}$
 reveal conformational changes during interfacial activation. Nat Struct Biol.

[bib1.bib102] van den Berg B, Tessari M, Boelens R, Dijkman R, Kaptein R, de Haas GH, Verheij HM (1995). Solution structure of porcine pancreatic phospholipase A
${}_{2}$
 complexed with micelles and a competitive inhibitor. J Biomol NMR.

[bib1.bib103] van der Horst MA, van Stokkum IH, Crielaard W, Hellingwerf KJ (2001). The role of the N-terminal domain of photoactive yellow protein in the transient partial unfolding during signalling state formation. FEBS Lett.

[bib1.bib104] van der Waals J, Hilbers K (2004). De geschiedenis van de scheikunde in Nederland 3, De ontwikkeling van de chemie van 1945 tot het begin van de jaren tachtig.

[bib1.bib105] van Gunsteren WF, Kaptein R, Zuiderweg ERP, Olsen WK (1984). Proceedings of the NATO/CECAM Workshop on Nucleic Acid Conformation and Dynamics.

[bib1.bib106] van Leeuwen PWNM, Kaptein R, Huis R, Kalisvaart WI (1975). CIDNP Studies of Reactions of Alkyllead compounds with Hexachloroacetone and Hexachlorocyclopentadiene. J Organomet Chem.

[bib1.bib107] Vis H, Mariani M, Vorgias CE, Wilson KS, Kaptein R, Boelens R (1995). Solution structure of the HU protein from *Bacillus stearothermophilus*. J Mol Biol.

[bib1.bib108] von Hippel PH, Berg OG (1989). Facilitated target location in biological systems. J Biol Chem.

[bib1.bib109] Vuister GW, Boelens R (1987). Three-dimensional 
J
-resolved NMR spectroscopy. J Magn Reson.

[bib1.bib110] Vuister GW, Boelens R, Kaptein R (1988). Non-selective three-dimensional NMR spectroscopy. The 3D NOE-HOHAHA experiment. J Magn Reson.

[bib1.bib111] Ward HR, Lawler RG (1967). Nuclear magnetic resonance emission and enhanced absorption in rapid organometallic reactions. J Am Chem Soc.

[bib1.bib112] Zetta L, Kaptein R, Hore PJ (1982). A Photo-CIDNP investigation of tyrosine mobility and exposure in human beta-endorphin in the presence of phospholipid micelles. FEBS Lett.

[bib1.bib113] Zetta L, Böhmer V, Kaptein R (1988). Rapid hydrogen atom transfer in oligophenols. A photo-CIDNP study. J Magn Reson.

[bib1.bib114] Zuiderweg ERP, Fesik SW (1989). Heteronuclear three-dimensional NMR spectroscopy of the inflammatory protein C5a. Biochemistry.

[bib1.bib115] Zuiderweg ERP, Kaptein R, Wüthrich K (1983). Secondary structure of the lac repressor DNA-binding domain by two-dimensional 1H nuclear magnetic resonance in solution. P Natl Acad Sci-Biol.

[bib1.bib116] Zuiderweg ERP, Kaptein R, Wüthrich K (1983). Sequence-specific resonance assignments in the 
${}^{1}H$
 nuclear-magnetic-resonance spectrum of the Lac repressor DNA-binding domain 1–51 from *Escherichia coli* by two-dimensional spectroscopy. Eur J Biochem.

[bib1.bib117] Zuiderweg ERP, Scheek RM, Boelens R, van Gunsteren WF, Kaptein R (1985). Determination of protein structures from nuclear magnetic resonance data using a restrained molecular dynamics approach: The lac repressor DNA binding domain. Biochimie.

